# On the Design of Smart Parking Networks in the Smart Cities: An Optimal Sensor Placement Model

**DOI:** 10.3390/s150715443

**Published:** 2015-06-30

**Authors:** Antoine Bagula, Lorenzo Castelli, Marco Zennaro

**Affiliations:** 1 Intelligent Systems and Advanced Telecommunication Laboratory, Department of Computer Science, University of the Western Cape, Private Bag X17, Bellville 7535, Cape Town, South Africa; E-Mail: bbagula@uwc.ac.za; 2 Dipartimento di Ingegneria e Architettura, Università degli Studi di Trieste, Via A.Valerio 10, 34127 Trieste, Italy; E-Mail: castelli@units.it; 3 T/ICT4D Laboratory, The Abdus Salam International Centre for Theoretical Physics, Via Beirut 7, 34151 Trieste, Italy; E-Mail: mzennaro@ictp.it

**Keywords:** Internet-of-Things, wireless sensor networks, smart parking, radio frequency identification, optimal sensor placement

## Abstract

Smart parking is a typical IoT application that can benefit from advances in sensor, actuator and RFID technologies to provide many services to its users and parking owners of a smart city. This paper considers a smart parking infrastructure where sensors are laid down on the parking spots to detect car presence and RFID readers are embedded into parking gates to identify cars and help in the billing of the smart parking. Both types of devices are endowed with wired and wireless communication capabilities for reporting to a gateway where the situation recognition is performed. The sensor devices are tasked to play one of the three roles: (1) slave sensor nodes located on the parking spot to detect car presence/absence; (2) master nodes located at one of the edges of a parking lot to detect presence and collect the sensor readings from the slave nodes; and (3) repeater sensor nodes, also called “anchor” nodes, located strategically at specific locations in the parking lot to increase the coverage and connectivity of the wireless sensor network. While slave and master nodes are placed based on geographic constraints, the optimal placement of the relay/anchor sensor nodes in smart parking is an important parameter upon which the cost and efficiency of the parking system depends. We formulate the optimal placement of sensors in smart parking as an integer linear programming multi-objective problem optimizing the sensor network engineering efficiency in terms of coverage and lifetime maximization, as well as its economic gain in terms of the number of sensors deployed for a specific coverage and lifetime. We propose an exact solution to the node placement problem using single-step and two-step solutions implemented in the Mosel language based on the Xpress-MPsuite of libraries. Experimental results reveal the relative efficiency of the single-step compared to the two-step model on different performance parameters. These results are consolidated by simulation results, which reveal that our solution outperforms a random placement in terms of both energy consumption, delay and throughput achieved by a smart parking network.

## 1. Introduction

As a typical component of a smart city application, smart parking is a good illustration of how the Internet-of-Things (IoT) will be pervasively deployed in our daily living environments to provide different services to different users. It can be used for example to enable a driver to access a smart city application through a mobile device or the Internet in order to locate and reserve a parking spot in a city's airport based on some preferences (e.g., close to the airport international departure/arrival) and prepay for the parking spot using his credit/debit card. Similarly, a citizen of a smart city may use his mobile device, a tablet or the Internet to access from home the smart city application to (1) find a free parking spot in the city center; (2) be advised of the probability for the parking spot to be still available upon his arrival in the city center and (3) decide on reserving and prepaying for such a parking spot. When deployed in a parking lot, a smart parking system can provide efficient car parking management through (1) remote parking spot localization and reservation; (2) fast car retrieval; (3) parking regulation using gate control and management; (4) car security and protection in the parking lot by associating car movement to a specific RFID tag; (5) parking gate management and many other services, such as parking billing and payment by replacing the paper-based ticketing by RFID tags.

Car parking management has been addressed using two models: parking gate monitoring and parking lot monitoring. Building upon an account of the entries/exits to/from the parking lot, smart parking management based on gate monitoring may provide important services for drivers, such as the capability of checking the availability of a free spot, reservation over the Internet, *etc.* In smart parking management based on parking lot monitoring, each parking spot is equipped with a sensor (camera or presence sensor) to detect the presence/absence of vehicles with the objective of building an availability map that can be used for parking guidance, reservation and the other services described above when combined with RFID identification devices. While the gate monitoring model results in simple and lower cost parking systems, the lot management model is more expensive, but provides many more services to the drivers and the parking lot owner. Smart parking management using lot monitoring can be classified into multi-parking management used to manage different parking lots in different indoor and outdoor areas or mono-parking management generally targeting indoor parking lots and focusing on a single parking lot's management. The focus of this work lies on mono-parking management and targets the evaluation of the economic and engineering efficiencies of a smart parking system using hybrid wired/wireless communication.

### 1.1. Related Work

The authors of [1–3] propose smart parking managers using RFID technology and embedded systems with the objective of helping a user check the availability of a parking space over the Internet. The authors of [4,5] propose a system that uses image detection. In [6–8], a mono-parking wireless sensor network is described where wireless sensors are deployed in a car parking lot, with each parking spotequipped with one sensor node. The authors of [9,10] propose a multi-parking system to extend the services to a scaler larger than a single car parking lot; for instance, outdoor car parking management, e.g., in streets, guiding towards an appropriate car parking lot within a city. This may require a collaboration between multiple car parking managers. In [11], the authors propose KATHODIGOS, which is a smart parking system that use wireless technologies and wireless applications to acquire information about the status of roadside parking spaces. The obtained information is transmitted to a central information system through gateways. The solution in [12] proposes a framework of parking management, called iParking, that monitors incoming/outgoing vehicles using a sensor network. iParking calculates the number of available parking spaces and disseminates the information to the parking lot's customers. In [13], the authors describe a street parking system based on a wireless sensor networks. The system can monitor the state of every parking space by deploying a magnetic sensor node on the space. A vehicle detection algorithm is proposed to accurately detect a parking car, and adaptive sampling is used to reduce the energy consumption. Nawaz *et al.* [14] present ParkSense, a smartphone-based sensing system that detects a driver vacating a parking spot. Most of the solutions proposed so far consider the use of either WSN or RFID and focus on the engineering of the traffic, discounting the engineering of the network through an efficient placement of sensors in the parking lot.

Starting with a large body of research devoted to the base station (BS) location problem, the optimal placement of nodes in a mesh network design has evolved towards a wireless sensor network topology optimization with solutions ranging from dynamic programming [15] to genetic algorithms [16,17] and Tabu search [18]. Borrowed from the operations research field, the BS location problem is part of the larger area of facility location, where a set of demand points must be covered by a set of facilities with the objective of optimizing a given objective, such as the total distance between demand points and their closest facility, subject to some constraints. This is closely related to the maximal covering location problem (MCLP) [19,20], also referred to as a location-allocation problem in WSN, where as many demand points as possible must be covered with p sensors of a fixed radius.

Looking at WSN engineering issues for the emerging IoT, researchers have proposed several solutions to achieve optimal node placement through topology control. Focusing on sensing coverage maximization with little or no attention to the communication requirement between sensors, the works presented in [21–26] are similar to the BS location problem, as they focus on optimizing the location of the sensors in order to maximize their collective coverage. While [22] used integer programming, the works in [21,23–25] are built around different greedy heuristic rules to incrementally deploy the sensors. The technique used in [26] is adapted from virtual force methods for the sensor deployment.

By revealing optimal ways of connecting sensors through resolution of an optimal placement problem, layout optimization studies, such as those provided in [27–31], may also reduce energy consumption. While [27,29] are based on a heuristic solution using multi-objective evolutionary optimization, the placement problem in [31] is formulated as an non-linear optimization problem solved using a self-incremental algorithm that adds nodes one-at-a-time into the network in the most efficient identified way. In [30], the optimal placement of static sensors in a network is used to help an agent navigate in an area by using range measurements to the sensors to localize itself. Aiming to study different regular grid positioning methods for sensor networks, [32] deduces in each case (square, triangular and hexagonal) the minimum number of sensors required to provide full coverage, and the resulting fault-tolerance is expressed in the minimum number of nodes that have to be shut down in order to degrade the network coverage. Looking at the positioning of sensors in a terrain from the point of view of data transmission, the work presented in [33] divides the studied terrain into cells, then analyses how N sensors should be distributed among the cells, in a way that avoids network bottlenecks and data loss. Two greedy algorithms selecting the locations for a sensor network with a minimal number of nodes using a grid model for the terrain and a probabilistic coverage model for the sensors are presented in [34]. Building upon geospatial constraints, the model allows the inclusion of the effect of obstacles and terrain height and incorporation of an importance factor giving preference to the coverage of some part of the terrain.

### 1.2. Contributions and Outline

While much effort has been invested into smart parking lot management with a specific focus on traffic engineering of the sensor readings from the parking spots to the gateway, the optimal placement of sensor devices in a parking lot to increase coverage and connectivity in a cost-effective way is an important parameter upon which the efficient parking management depends. This paper revisits the problem of node placement in wireless sensor networks with the objective of maximizing the coverage and lifetime of the network when planning a mesh sensor network for a smart parking system. We formulate the optimal node placement as a multi-objective optimization problem and build upon a preemptive topology control approach to propose and evaluate the performance of an exact solution model to the problem. Using a mathematical formulation that is based on integer linear programming, we propose single-step and two-step solutions, both implemented in the Mosel language based on the XPress-MPsuite of libraries. Using Mosel optimizations, we also propose implementation improvements leading to finding the best number of nodes optimizing our objective function in the minimum computation time. This solution is referred to in our paper as the “optimized” solution as compared to a “static” solution that finds the solution in more computation runs. Experimental results reveal the relative efficiency of the single-step compared to the two-step model on different performance parameters. In contrast to many studies that use simplified models, which are based on only the communication range to guide the optimal placement of sensors in the sensing environment, our model integrates the sensing range as an important parameter that can impact not only the engineering, but also the economic efficiency of a smart parking system.

The remainder of this paper is as follows. Section 2 presents the smart parking system upon which our model is built and highlights some of the practical requirements and issues associated with such a parking system model. Section 3 describes our proposed multi-objective optimization model in terms of its mathematical formulation, including the decision variables, the objective function, the constraints, as well as a brief overview of the exact solver. Starting by a description of the main performance parameters used in our experimentation, Section 4 presents the environmental setting, as well as the experimental results obtained for both the single- and two-step algorithms using the static and the optimized solutions. Our conclusion and future work are presented in Section 5.

## 2. The Smart Parking System

The smart parking model considered in this paper builds upon the real parking prototype proposed in [35], which was tested in an outdoor parking lot at the CERISTresearch center in Algiers, Algeria.

### 2.1. The Smart Parking Prototype

**Figure 1:**
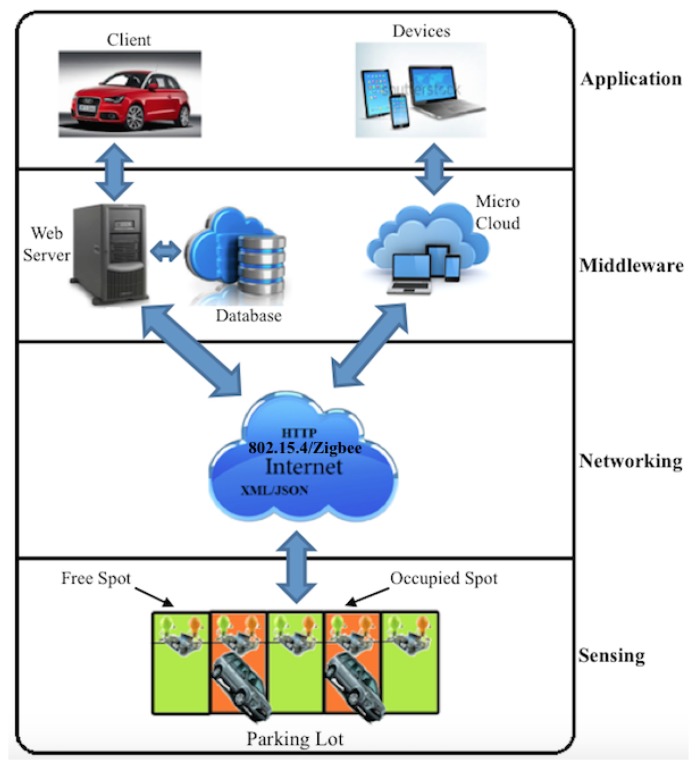
The smart parking system.

The smart parking system (SPS) considered in this paper was built around a sensor-based method and designed based on a multi-layer framework to enable modularity and scalability and to provide different services to different users of a parking system. The framework depicted by Figure 1 includes four layers: a sensing, networking, middleware and application layer.

#### 2.1.1. The Sensing Layer

This layer defines a platform where sensor devices are embedded into the parking lot to detect car presence/absence, and RFID devices located at the parking gates and strategic points of the parking are used to identify cars based on a unique mapping between RFID tags and cars. Three types of sensor devices are used: (1) slave devices, also called “receivers”, which are placed on the parking spots to detect presence/absence; (2) master devices, also called “transmitters”, which are tasked to collect the sensor readings from their connected slave devices and transmit these readings to a gateway for further processing; and (3) “anchor” devices used as repeaters to increase the wireless sensor coverage of the parking for efficient routing of the sensor readings. The slave devices are connected to the master devices through wired communication using the I2Cprotocol. For sensing purposes, micro-controllers equipped with ultrasonic sensors were used, and an ARM-based Panda board was used as the gateway.

#### 2.1.2. The Networking Layer

Different modes of communication have been proposed in this layer to support communication from the master and anchor sensors to the sensor gateway and from the gateway to the parking users (parking drivers, remote users and parking owners). These include (1) the 802.15.4/ZigBee communication for routing the sensor readings from master sensors to the gateway, (2) TCP/IP over Ethernet for connecting the gateway to the parking server and database and (3) Internet access for remote access to the smart parking system from outside.

#### 2.1.3. The Middleware Layer

This layer is the layer where situation recognition is performed using intelligent algorithms and efficient visualization techniques to present smart services and a user-friendly interface to the users. This layer hosts different databases and associated servers and manages all of the software intelligence provided by the smart parking system to provide smart services to users by enabling communication between the application layer where services are requested and the lower layers where smart devices are embedded into the parking lot to provide smart services.

#### 2.1.4. The Application Layer

The application layer is the layer where the different services are defined and provided to different users. Client devices have been connected via the TCP/IP protocol to a parking database. The latter is updated in real time with the status of the parking lots. Two kinds of client applications have been considered for parking lot monitoring: (1) a mobile device application for phones and tablets; and (2) a desktop application for laptops and desktop computers.

### 2.2. The Smart Parking Services

The different services provided by our smart parking system include:

- Proactive guidance service: In our current implementation, active RFID technology has been used to replace the paper-based ticketing system and provide drivers' guidance by enabling car drivers to use their RFID tag in order to be guided toward their pre-allocated parking spot. In this case, RFID readers placed at strategic points of the parking lot are used to get the tag's ID and to interrogate a database managed by the parking manager to retrieve the pre-allocated parking spot and guide the driver. Each vehicle driver is assumed to have been given an active RFID tag.
- Reactive guidance service: When parking spot pre-allocation is not an option, a reactive guidance model is used where the parking RFID readers are programmed to retrieve the nearest available (optimal) parking spot from the parking manager. The optimal location is calculated based on the current location of the car and a map of free spots, which is built based on the car presence/absence detection system and maintained by one of the parking servers.
- Car retrieval service: This service also uses the tag carried by the driver to interrogate the readers located in different spots and to transmit the tag's ID to the parking manager. The latter checks the occupied parking spots in the database and in the parking lot and returns a response to the parking guidance node for visualization using a visualization management system (VMS).
- Smart parking regulation: The smart parking regulation takes place when the car drivers have a specific parking area pre-allocated for their use or when parking spot reservation is implemented. When a car is detected in a parking spot, a presence event, including the RFID tag's ID of a car and its location, is transmitted to the parking manager. This latter uses the information received to check in its database if the identified car is authorized to access the location.
- Parking security services: The current implementation of the SPS enables a driver to be provided either a permanent RFID tag in the case of a private parking spot or a temporary one at the parking entrance in the case of a public parking spot. The tag is used for different purposes, including security purposes through (1) a one-to-one mapping between the RFID tag identifying a car and a parking spot and (2) a security check process where the tag is used to detect car theft when the parking manager software detects a car leaving the parking spot while its corresponding tag is signaled by the closest reader to be far from the identified parking spot.

While the first version of our prototype includes the services described above, many other services are planned to be included in our SPS in the near future. These include:

- Parking availability status by integrating into the car detection system sources of light on parking spots, which are controlled by actuators to inform of the status of a parking spot: e.g., red for occupied, green for empty, yellow for reserved and blue for out of service.
- Parking payment service to replace the paper-based payment by RFID tags, which are collected at the parking entrance and used when leaving the parking lot at an RFID-based payment machine to compute the time spent in the parking lot and subsequently bill the car driver.
- Parking gate management to enable gates to be opened automatically to enable an authorized driver access to a specific part of the the parking lot. This service is similar to the smart parking regulation service, as they are both built around an authorization system aimed to constrain access based on permissions.
- Remote availability checking using the Internet and/or the GSM network to check in real time the availability of the smart parking system. By enabling access to a smart parking availability status “anytime” and “anywhere”, this service can provide substantial time savings for a parking user.

### 2.3. Implementation Challenges

The design and implementation of the smart parking system described above has raised several challenges. Some have been addressed during the prototype implementation at CERIST, while others have been reserved for future research work.

- Sensor selection: The selection of the appropriate sensing technology is a challenging issue upon which the efficiency of a smart parking deployment depends. Size, reliability, adaptation to environmental changes, robustness and cost are among the parameters that usually play a key role when selecting the type of sensors for a smart parking system. Furthermore, intrusive sensors, which need to be installed directly on the pavement surface, are usually avoided, as they have a number of challenges, including the need for digging and tunneling under the road surface. Ultrasonic sensors were considered in our deployment, as they were low cost and required simple installation by being fixed on the ground.
- I2C protocol issues: One of the main challenges was to alter the I2C for our application, given that the it has not been designed to operate at long distances. Some changes to some I2C parameters have been necessary, such as the pull resistance and clock frequency.
- Power supply issues: Another challenge was the power supply connection for all of the sensor motes with efficient power consumption.
- Potential imperfections: When deployed outdoors, ultrasonic sensors are known to be sensitive to temperature changes and extreme air turbulence. Potential imperfections associated with indoor wireless communication, such as interference, reflection, diffraction and wall penetration, are challenges that will have to be dealt with when deploying the SPS indoor.
- Efficient wireless coverage: For both indoor and outdoor deployment, the efficient wireless coverage is an important parameter upon which the lifetime of the smart parking system depends. This is the main issue addressed in this paper.

## 3. The Optimal Sensor Placement Model

**Figure 2:**
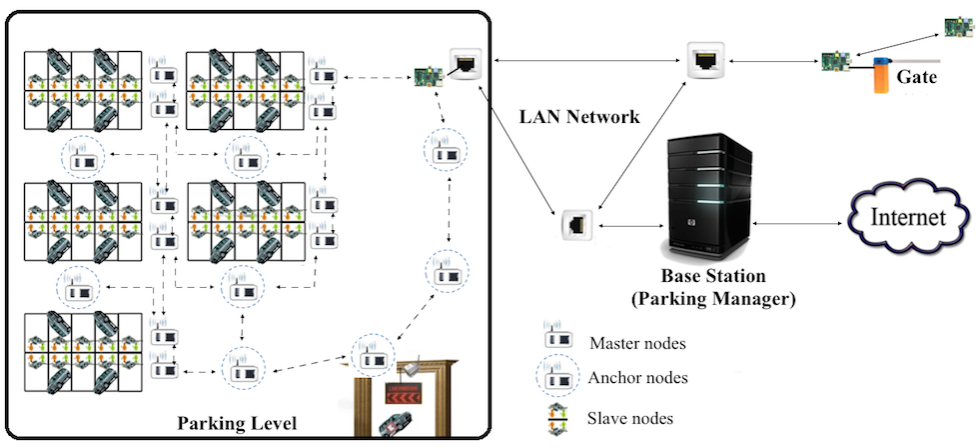
The smart parking sensor placement.

Figure 2 depicts the layout of a smart parking system where sensors are organized into islands of heterogeneous devices (slave sensors, master sensors and repeater/anchor sensors), which are interconnected by either wired links using I2C and Ethernet protocols or wireless links operating the 802.15.4/Zigbee protocol. In such a heterogeneous environment, the slave sensor nodes are located on the parking spots to detect car presence/absence and report to a master sensor node, while the master sensor nodes are located at the edge of the parking lot and tasked to report the sensor readings to the gateway where further processing is performed.

The master and slave sensors are located in the parking lot based on geolocation constraints, which can lead to poor coverage and the inability for the sensor readings to reach the gateway located at the entrance of the parking lot. Repeater nodes, also referred to as “anchor” nodes, are integrated into the smart parking system to mitigate this issue by strategically placing these sensors at optimally-computed locations to increase the sensor network coverage and connectivity. As deployed in the smart parking lot, the slave and master nodes form the edge of the wireless sensor network, while the anchor nodes constitute the backbone of the sensor network.

### 3.1. A Multi-Objective Optimization Problem

The optimal placement of anchor nodes in the smart parking system may impact not only the system's engineering efficiency in terms of efficient data collection and on-time delivery to the gateway, but also the economic gain expressed in terms of the number of sensors that are required for monitoring the parking area, as this reflects the financial cost of the deployment. As generally formulated, the optimal node placement in networks consists of determining the position of nodes that minimizes/maximizes a pre-defined penalty/reward function subject to a set of specifications and/or constraints for a given deployment area to be covered. The optimal node placement has been widely applied to both base station (BS) location in cellular networks and sensor placement in wireless sensor networks. However, sensor network and BS placement problems differ in many aspects, since, while the BS placement problem does not require BS connectivity, the optimal sensor placement requires connectivity between sensors to allow multi-hop dissemination of the readings of all sensors to a unique gateway, where further processing is performed, as suggested by the traditional one-to-m sensor deployment model.

While many works have resulted in simplifications leading to single objective formulations, the optimal node placement in a WSN is widely known as a multi-objective optimization problem involving competing objectives, such as coverage and lifetime maximization, with coverage maximization degrading lifetime performance, while lifetime maximization reduces WSN coverage. This can be illustrated by the typical “conference room”, scenario where the more close to each other the participants are, the less energy they will spend for communication (they are maximizing the lifetime of the meeting), while being close to each other results in covering less space in the room (minimize coverage). Inversely, though increasing coverage, scattering the conference participants in a room by having longer average distance between them leads to higher energy consumption for communication (reduction of the lifetime of the meeting). In a mathematical formulation for node placement in WSN, “scattering” translates into coverage maximization, while “closeness” is mapped to lifetime maximization expressed by distance minimization or equivalently min used distance maximization. Furthermore, as suggested by [29], deploying sensors based on a single objective may lead to problems, such as (1) a disconnected network when the sensing range is of the same order or larger than the communication range, as is the case for seismic sensors; and (2) a partition of the WSN if the areas to be covered are disjoint.

### 3.2. Mathematical Formulation

The optimal placement of anchor devices is a network engineering problem that can be solved using the mixed integer linear formulation proposed in this paper to determine the position of a set of *N_S_* sensors and one sink, such that the sensing coverage and the WSN lifetime are maximized. The area to be covered (the parking lot) is represented as a square of *LxL* cells. We assume that any cell is either completely covered or not covered at all, *i.e.*, partial coverage of a cell is not allowed. Each sensor covers all of the cells falling within the sensing range *S_R_* and communicates with all other sensors (or the sink) located within the communication range *C_R_*. As already mentioned, the WSN lifetime depends on the distance between sensor nodes and between sensor nodes and the sink: the shorter the distance, the longer the lifetime, and *vice versa.* We also assume that the WSN network is connected, *i.e.*, each sensor should be within the communication range of another sensor, and the sink should be within the communication range of at least one sensor. The distance between two cells is calculated as the Euclidean distance.

The model's formulation requires the following definitions of the coordinates of each cell. Let *N* = *LxL* be the number of available cells. The coordinates *xc*(*i*) and *yc*(*i*) of the *i*-th cell, *i* = 1,…,*N* are:

$$\begin{array}{ll} xc(\text{i}) & =\{\begin{array}{cc} L & \text{if}\;(\text{i MOD L})=0\\ \text{i MOD L} & \text{otherwise} \end{array}\\ yc(\text{i}) & =\{\begin{array}{cc} \text{i DIV L} & \text{if}\;(\text{i MOD L})=0\\ (\text{i DIV L})+1 & \text{otherwise} \end{array} \end{array}$$

Hence, the Euclidean distance between each possible cell is:

$$d_{ij}=\sqrt{{\left[xc(i)-xc(j)\right]}^{2}+{\left[yc(i)-yc(j)\right]}^{2}}$$

#### Decision Variables

$$\begin{array}{ll} x_{\text{i}} & =\{\begin{array}{cc} 1, & \text{if a sensor is located at cell}\;i;\\ 0, & \text{otherwise}. \end{array}\\ s_{i} & =\{\begin{array}{cc} 1, & \text{if the sink is located at cell}\;i;\\ 0, & \text{otherwise}. \end{array}\\ y_{i} & =\{\begin{array}{cc} 1, & \text{if cell}\;i\;\text{is covered},i.e.,\text{it falls within the sensing range of a sensor};\\ 0, & \text{otherwise}. \end{array}\\ z_{\mathrm{ij}} & =\{\begin{array}{cc} 1, & \text{if a sensor is at cell}\;i\;\text{and another one}(\text{or the sink})\text{is at cell}\;j\text{and they are within the communication range},i.e.,d_{\mathrm{ij}}\leq C_{R};\\ 0, & \text{if cells}\;i\;\text{and}\;j\;\text{are within the communication range and at least one cell is empty}. \end{array} \end{array}$$

If cells *i* and *j* are too far away, *i.e.*, farther than *C_R_*, a link between them cannot be established in any case. Hence, the corresponding decision variables are not created (this action allows one to reduce the number of decision variables and constraints to be used in the model).

#### Objective function

Relying on the above notation and definitions, the objective function can be expressed as the sum of the covered cells minus the total distance among all sensors and sinks:

$$\max\left\{\sum_{i=1}^{N}y_{i}-\sum_{i=1}^{N}\sum_{j=1}^{N}d_{ij}\cdot z_{ij}\right\}$$

Note that:

- As expressed above, the optimization objective is a mixed function combining a reward for covering the given area (the sum of the covered cells) and a penalty for scattering the sensor nodes and, thus, reducing the WSN lifetime (the min used total distance).
- Though the two objectives might be weighted differently, they have been assigned equal weight in our mixed objective function to express an equal importance allocated by the WSN designer to coverage and lifetime.

#### Constraints

The model's constraint set is as follows:

(1) $$\sum_{i\in N}x_{i}=N_{S}$$

(2) $$\sum_{i\in N}s_{i}=1$$

(3) $$\begin{array}{cc} x_{i}+s_{i}\leq 1 & \forall i\in N \end{array}$$

(4) $$\begin{array}{cc} x_{i}\leq y_{j} & \forall i,j\in N|d_{ij}\leq S_{R} \end{array}$$

(5) $$\begin{array}{cc} y_{i}\leq \underset{j\in N|d_{ij}\leq S_{R}}{\text{∑}}x_{j} & \forall i\in N \end{array}$$

(7) $$\begin{array}{cc} x_{i}+s_{i}\leq \underset{j\in N|d_{ij}\leq C_{R}}{\text{∑}}x_{j} & \forall i\in N \end{array}$$

(8) $$\begin{array}{cc} \begin{array}{cc} z_{ij}\leq x_{i} & \forall i,j\in N|j>i \end{array} & \text{AND} \end{array}\;d_{ij}\leq C_{R}$$

(9) $$\begin{array}{cc} \begin{array}{cc} z_{ij}\leq x_{j}+s_{i} & \forall i,j\in N|j>i \end{array} & \text{AND} \end{array}\;d_{ij}\leq C_{R}$$

(10) $$\begin{array}{cc} \begin{array}{cc} z_{ij}\geq x_{i}+x_{j}+s_{j}-1 & \forall i,j\in N|j>i \end{array} & \text{AND} \end{array}\;d_{ij}\leq C_{R}$$

(11) $$\begin{array}{cc} x_{i}\leq \underset{j\in N}{\text{∑}}z_{ij} & \forall i\in N \end{array}$$

(12) $$\begin{array}{cc} s_{i}\leq \underset{j\in N}{\text{∑}}z_{ji} & \forall i\in N \end{array}$$

Constraints (1) and (2) ensure that all available sensors and the sink are placed in the area under consideration. Constraint (3) imposes that in each cell, at most one sensor or one sink can be placed. The cell coverage is implemented through Constraints (4) and (5). In fact, if there is a sensor in cell *i*, then all cells at a distance lower or equal to *S_R_* are covered (constraint 4). On the other hand, if a cell is covered, then there must exist a sensor within distance *S_R_* (Constraint (5)). The following constraints build the network. In particular, if a sensor or a sink is in cell *i*, there must be a sensor within distance *C_R_* (Constraint (7)). Constraints (8)–(10) implement the AND function that, in the case that the distance is lower or equal to *C_R_*, establishes a link (*i.e.*, *z_ij_* = 1) if and only if there is a sensor in *i* and a sensor or the sink in *j*. We may notice that Constraints (8)–(10) are defined only for *j* > *i*, *i.e.*, if there is a sensor in both cells *i* and *j*, only the link from cell *i* to cell *j* is created, if *d_ij_* ≤ *C_R_*, and not from *j* to *i*. Thus, this formulation naturally induces an orientation to all arcs, such that a sensor in cell *i* passes its information only to sensors located at cells *j* higher than *i*. In this way, the number of decision variables is dramatically reduced without loss of generality of the optimal solution. It also follows that in the sink, at least one link has to arrive, and no links must depart from it (Constraint (12)). Thus, among all cells occupied either by a sensor or the sink, the sink is located in the highest position. Since the sink does not have sensing capabilities, the optimal coverage does not depends on the sink position, and thus, this formulation allows one to eliminate equivalent solutions with respect to the sink position. Finally, Constraint (12), together with Constraint (11) (at least one link departs from each sensor), ensures the connectivity of the network.

### 3.3. An Adaptive Optimization Model

While the model presented above uses a static approach that consists of finding a fixed number of sensors *N_s_* as expressed by Constraint (1):

$$\sum_{i\in N}x(i)=N_{S}$$

which places exactly *N_S_* nodes in the grid, an adaptive model can be obtained by changing this constraint into the constraint:

$$\sum_{i\in N}x(i)=N_{S}$$

which requires the number of nodes *N_S_* to be large enough to meet the sensor placement constraint. Such a change will lead to a model that will adaptively/automatically find the best number of sensors that maximize our objective function. It is expected that this will lead to finding the best configuration in fewer runs of the algorithm.

### 3.4. The Exact Model Solver

As expressed above, the mathematical model is an integer linear programming problem that can be solved using either exact methods or approximative methods based on heuristics, such as those borrowed from the evolutionary computation field [36].

Building on the Mosel language widely used to solve linear, integer and quadratic programming problems based on the the Xpress-Optimizer library [37], we propose two different solution models for the multi-objective optimization problem: (1) a single-step solution that considers a multi-constrained single objective optimization problem that integrates the coverage and lifetime objectives into one objective subject to the constraints as expressed by the mathematical formulation above; and (2) a two-step or lexicographic solution, where one of the objectives (maximum coverage) is solved first to find a solution space that is searched for a solution that solves the second objective (minimum distance or maximum lifetime). As currently implemented, the Xpress suite includes extension modules that provide access to the other solvers, such as (1) a nonlinear programming solver named “Xpress-SLP”; (2) a stochastic modeling engine referred to as “Xpress-SP” and (3) a constraint programming solver called “Xpress-Kalis”.

## 4. Performance Evaluation

**Figure 3:**
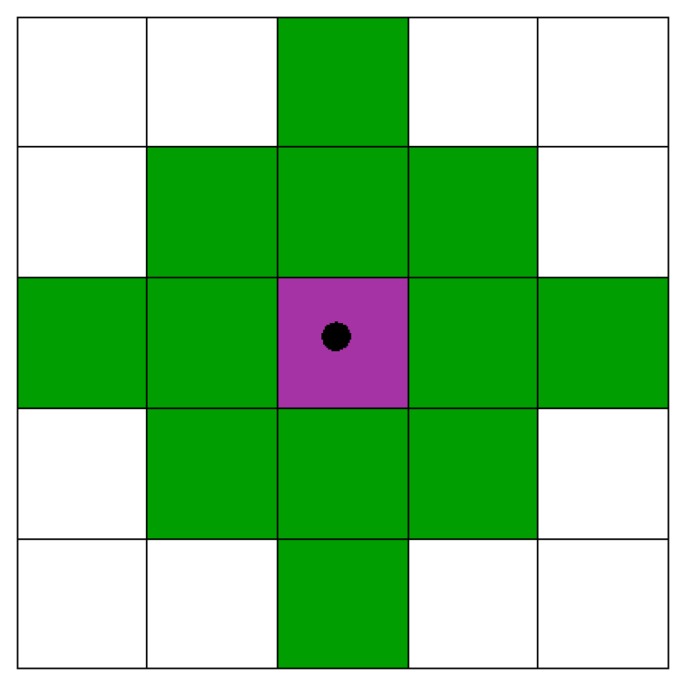
The sensor placement model.

We conducted a set of experiments to analyze the case of a squared shape area of 10 × 10 cells to be optimally covered by a variable number of sensors with the objective of maximizing coverage and WSN lifetime as expressed by the model formulation in Section 2. Our expectation was to find an optimal number of anchor/repeater sensors in a parking lot that could increase the coverage and connectivity of a smart parking system where slave and master sensors have been placed based on application and geographic constraints. We set the sensing range *S_R_* equal to two in terms of Euclidean distance. Thus, each sensor can cover at maximum 13 cells. This is illustrated by Figure 3, where we can see that the green cells are those covered if there is a sensor located in the pink cell. It is also assumed that a sensor covers its cell. The communication range *C_R_* is set equal to four in terms of Euclidean distance.

We consider the case where the optimal placement has been designed for (1) a hybrid wired-wireless backbone for smart parking and (2) a wireless backbone for smart parking. In the hybrid deployment, a set of hybrid sensor/RFID nodes are placed in a smart parking lot to serve as a backbone for a wired sensor network, which uses I2C to link ultra-sonic sensors as slaves of the hybrid nodes. In the wireless deployment, a set of hybrid nodes are placed in the smart parking lot to serve as a backbone for sensor motes located on the parking spots.

### 4.1. Network Engineering Performance: Backbone Design

The main performance parameters considered in our experiments consisted of:

- *MaxCov:* the theoretical maximum coverage, *i.e.*, *N_S_* * 13. If this number is higher than 100, it should be set to 100 to express maximal coverage. The higher MaxCovis, the better the solution is. Higher MaxCov is an indication of efficiency, while lower values of this parameter reveal poor performance.
- *CovCells:* the number of covered cells by the optimal solution. This is an expression of the coverage that looks at the number of cells rather than the surface covered. An algorithm that covers more cells (higher CovCells) will be considered as more efficient than one that covers a lower number of cells (lower CovCells).
- *Dist:* the total distance between sensors (and sink) in the optimal solution. It is an expression of the WSN lifetime that reveals the energy consumption, since communication over a longer distance leads to much higher energy depletion than over a shorter distance. A WSN operating over a shorter total distance will have a longer lifetime than one operating over a longer total distance. Therefore, lower *Dist* values are an indication of performance improvement.
- *OBJ: CovCells-Dist:* the optimal solution that needs to be maximized. Note that *Dist* is min used in this expression to express a penalty for communicating over a longer distance, while *CovCells* is plusedto express a reward for covering much more cells. In terms of network efficiency, higher values of *CovCells-Dist* will be preferred to lower values.
- *UB:* the best upper bound for the solution *OBJ*. By providing an upper bound, *UB* allows the placement of the real optimal solution between *OBJ* and *UB*.
- *GAP:* the gap expressing the maximum error you can have in taking *OBJ* as the optimal solution. It is given by (*UB*/*OBJ* − 1) * 100.
- *TIME:* the time needed to reach this gap. It expresses the efficiency of the solution in terms of processing overhead. A solution that computes faster is more efficient than one that computes longer.

These performance metrics are evaluated based on two main parameters: (1) *N* expressing the number of cells to be covered; and (2) *N_S_*, which is the number of sensors to be placed. All experiments were performed using Xpress Mosel 64-bit v3.2.0 and Xpress optimizer Version 20.00.11 on a Intel XEON with 16 CPUs at 2.27 GHz and with 16.00Gb of RAM.

#### 4.1.1. The Static Solution

Table 1 depicts the results obtained from a set of 15 experiments conducted using the single-step algorithm of the static solution. As implemented in the Xpress suite, the optimizer stops either when the gap is 1% or the computational time reaches 3600 s, whichever comes first. The same stopping criterion applies also in the following experiments. The table also reveals that we could reach the minimum gap only four times within the available time. These gaps are obtained for a 5-, 6-, 7- and 9-sensor topology.

The table also reveals that, as already mentioned, the optimal solution for a given instance of the problem lies between *OBJ*_1_ and *UB*_1_. We can see that from *N_S_* ≥ 15, *UB*_1_ is lower than an *OBJ*_1_ in some previous rows. As an example, when *N_S_* = 15, *UB*_1_ = 67.356, while when *N_S_* = 12, the value of *OBJ*_1_ is 69.111. Then, the optimal value for *N_S_* = 15 is certainly lower than the optimal value obtained in an earlier experiment with a lower number of sensors. This also applies for all cases with 16, …, 19 sensors. These results have two important implications:

- Technological implications: the number of sensors does not necessarily translate into performance gain, since placing more sensors in the area to be covered does not necessarily lead to a better solution. In our case, placing 12 sensors results in the optimal solution.
- Economic implications: as there exists a minimum number of placed sensors that lead to the optimal solution, designing an algorithm that finds such a minimal number in a minimum number of steps would result in a gain from an economic perspective, as the number of sensors used reflects the financial cost of the deployment and efficiency in terms of computation time used to find the optimal placement of sensors. This implication has resulted in the “adaptive” version of the algorithm previously described in Section 3.3 and discussed later in this section.

In the two-step case, we first maximize the coverage, and for this coverage, we minimize the distance. Table 2 shows the results (Table 2 has the same columns of Table 1).

If we compare the values of the objective functions and the time needed to reach them under the two optimization algorithms (single-step, two-steps), we have Table 3, where DiffSol(in %) = ((*OBJ*_1_/*OBJ*_2_) − 1) * 100 and DiffTime(in %) = ((*TIME*_1_/*TIME*_2_) − 1) * 100.

Table 3 reveals that for the static solution, the single-step algorithm reaches much better solutions (up to 44% higher), but is more computationally expensive. We also notice that in two cases (15- and 16-sensor placement), the two-step algorithm reaches a better solution than the one provided by the single-step algorithm. In both cases, as expected, the *OBJ*_2_ value lies between *OBJ*_1_ and *UB*_1_. Furthermore, when the number of sensors increases, the differences become smaller. However, as already mentioned, also in this case, it is useless to compute solutions for *N_S_* ≥ 15, as we see that better solutions can be reached with a lower number of sensors placement: *UB*_2_(*N_S_* ≥ 15) ≤ *OBJ*_2_(*N_S_* = 13). Overall, the single-step algorithm finds the best solution with a 12-sensor placement, whereas the two-step algorithm finds its best solution with a 13-sensor placement.

#### 4.1.2. The Adaptive Model

We conducted another set of experiments using the two algorithms (single-step and two-step) by fixing a bound to the number of nodes (a large number equal to 20 in our case) to find opportunistically the optimal configurations in terms of number of nodes found by the two algorithms. Thus, our formulation allows one to identify the best configuration both in terms of (1) the number of sensors and (2) the position of sensors with the objective of maximizing the trade-off between sensing coverage and lifetime expressed by the total distance.

Our experimental results presented in Table 4 revealed that in both cases, the best configurations result in 12 sensors in the single-step case and 13 sensors in the two-step case when using the same objective function value in both cases. These results are in agreement with the static solution, but with the advantage of a faster implementation requiring just one run per case.

The results depicted in Table 4 above reveal that using the two-step approach, we reach the optimal solution in much less time than the maximum allowed time (3600 s). On the other hand, the single-step algorithm provides better value of CovCells-Dist with 12 sensors. This appeals to a trade-off between the quality of the solution and computation time, which depends on the algorithm's use: while computational complexity is not a concern in the preemptive algorithms used during the planning phase of a WSN, it is a great concern in proactive algorithms that aim to repair a WSN configuration after planning. The study of such a tradeoff is beyond the scope of this paper.

**Figure 4:**
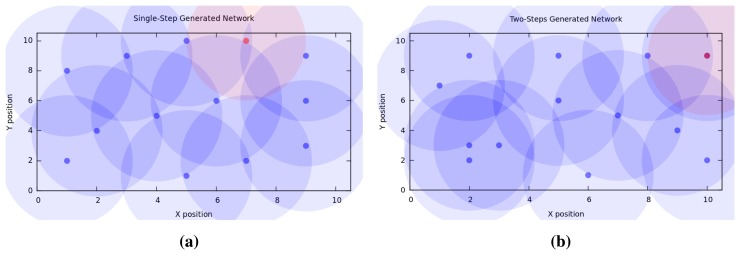
Optimized Topologies Comparison. (**a**) Single-step Generated Topology; (**b**) Two-steps Generated Topology.

Figure 4 depicts the two topologies generated by the optimized solution when using the single-step and two-steps algorithms. The figure reveals a difference not only in the sink (red dot) and normal node (blue dot) placement, but also a difference in the zones of possible interference depicted by the shaded areas.

### 4.2. Traffic Engineering Performance: Wireless Communication

Another set of experiments was conducted to evaluate the traffic engineering performance of the wireless backbone design using two types of network configurations: (1) a random network configuration designed using the K-connectivity algorithm to place the sensors in the network to achieve coverage and one-connectivity; and (2) an optimized network configuration designed using the single-step algorithm to generate a backbone network of anchor nodes for a set of pre-established slave and master nodes, as proposed in this paper. Following the modeling and performance analysis approach used in [38,39], we evaluated the performance of the two network configurations when using different routing algorithms to move the sensor readings from nodes to the sink node of the smart parking sensor network. These algorithms include (1) the energy-constrained multipath (ECMP) and the multi-constrained multipath (MCMP) routing algorithms; (2) the *ad hoc* on-demand (AODV) single-path algorithm and (3) the link-disjoint multipath routing (LDPR) algorithm.

#### 4.2.1. Performance Parameters

We considered three main performance parameters: (1) the average energy consumption for different delay requirements; (2) the average playback delay for different delay requirements; and (3) the average packet delivery for different delay requirements. The packet delivery is defined by the ratio of packets successfully delivered to the destination over the total number of transmitted packets. The path delay 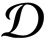
(*p*), that is the delay between a node and the sink, is given by the sum of link delays:

(13) $$D(p)=\sum_{\ell \in p}d_{\ell }$$

where *d_ℓ_* is the delay of data over the link *ℓ*. The playback delay is the delay taken to deliver the packets from one source to the sink (destination) in a multipath setting. It is the time taken to deliver the packets from the first one to the last one. It is mathematically defined by:

(14) $$D(P(s,d))=\underset{p\in P(s,d)}{\max}D(p)$$

where *P*(*s*, *d*) is the set of parallel paths between source *s* and destination *d*. Similarly, the path energy is expressed by the sum of the energies expanded over the links of the path. It is expressed by:

(15) $$W_{\ell }(p)=\sum_{\ell \in p}w_{\ell }$$

where *w_ℓ_* is the energy expanded on link *ℓ* expressed by:

(16) $$w_{\ell }=f_{\ell }\cdot w(s_{i},s_{i+1})$$

where *f_ℓ_* is the data rate on link *ℓ* and *w*(*s_i_*, *s_i_*_+1_) is the power required by the source node *s_i_* on link *ℓ* to receive a bit of data and then transmit it to the destination node *s_i_*_+1_. As defined by the energy model in [39], *w*(*s_i_*, *s_i_*_+1_) is expressed by:

(17) $$w(s_{i},s_{i+1})=\alpha_{1}+\alpha_{2}{\left\|x_{s_{i}}-x_{s_{i+1}}\right\|}^{n}$$

where *α*_1_ = *α*_11_ + *α*_12_ with *α*_11_ being the energy per bit consumed by *s_i_* as the transmitter, *α*_12_ the energy per bit consumed as the receiver and *α*_2_ accounting for the energy dissipated (link loss) during transmission. Typical values for *α*_1_ and *α*_2_ are, respectively, *α*_11_ = 180 nJ/bit and *α*_2_ = 10 pJ/bit/m^2^ for the link loss experienced by a radio transmission when *n* = 2 or *α*_2_ = 0.001 pJ/bit/m^4^ for the link loss experienced by a radio transmission when *n* = 4. *x_si_* and *x_si+1_* are the locations of the sensor nodes *s_i_* and *s_i_*_+1_, respectively, while ‖ *x_si_* − *x_si_*_+1_ ‖ is the Euclidean distance between the two sensor nodes.

#### 4.2.2. Average Energy Consumption

**Figure 5:**
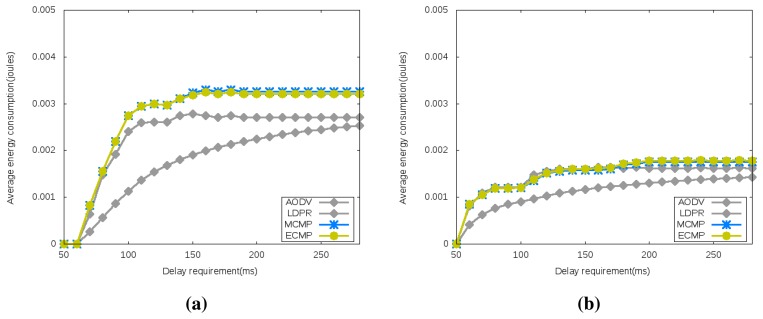
Average Energy Consumption. (**a**) Randomly Generated Configuration; (**b**) Optimally Generated Configuration.

Figure 5 reveals the energy consumption for both network configurations under different delay requirements. It reveals an energy consumption patterns with a steep increase followed by a plateau for all of the algorithms. The steep increase reveals a network operation zone where loosening the delay requirements leads to higher energy consumption, while the plateau reveals a delay threshold where further increases do not translate into equivalent energy consumption. The steep increase is in agreement with the findings in [38], where it was shown that the loosening of the delay constraints leads the multipath routing algorithms to find more paths for the traffic carried from nodes to the sink of the wireless sensor network. The results reveal that, in general, the optimally-generated network configuration results in lesser average energy consumption and, thus, longer sensor network lifetime.

#### 4.2.3. Average Playback Delay

**Figure 6:**
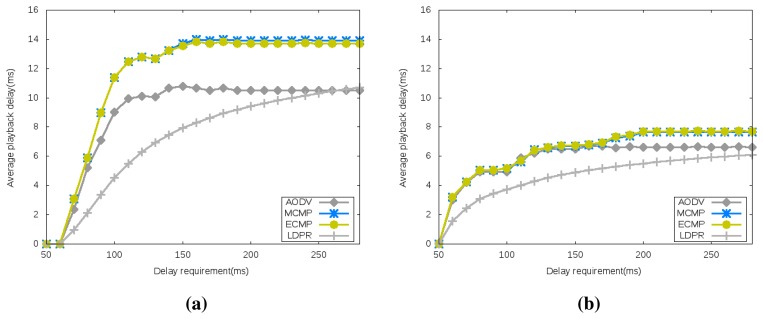
Average Playback Delay. (**a**) Randomly Generated Configuration; (**b**) Optimally Generated Configuration.

Figure 6 reveals the playback delay for both network configurations under different delay requirements. It reveals again the relative efficiency of the optimally-generated network configuration compared to the randomly generated one, as it leads to lower playback delays. Similarly to the energy performance, Figure 6 reveals a steep increase, where the playback delay increases with the delay requirements (loosening the delay requirements results in higher playback delay) followed by a plateau zone revealing a delay requirement threshold where further increases do not result in equivalent playback delay increases. This is in agreement with the results achieved in energy consumption, as in both cases, the loosening of the delay constraints leads to opening more and potentially longer paths for carrying the sensor readings; thus consuming more energy and potentially on longer paths with longer delays.

#### 4.2.4. Average Packet Delivery

**Figure 7:**
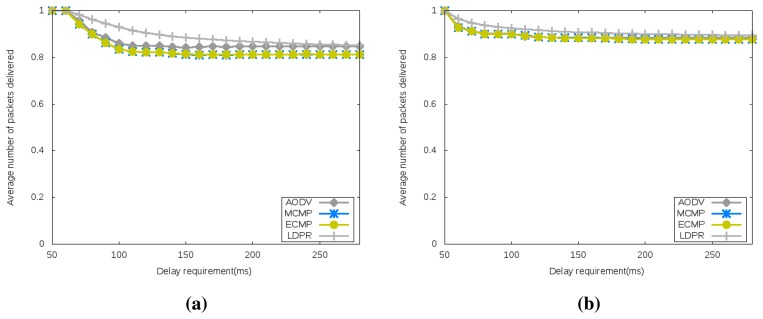
Average Packet Delivery. (**a**) Randomly Generated Configuration; (**b**) Optimally Generated Configuration.

As depicted by Figure 7, the average packet delivery is a measure of the throughput achieved by the two network topologies under different delay requirements. This figure shows that (1) the optimally-generated configuration leads to slightly better throughput and (2) the throughputs achieved by the different algorithms are similar.

## 5. Conclusions and Future Work

Building upon preemptive topology control, this paper presents and evaluates the performance of a multi-objective optimization model for optimal node placement in wireless sensor networks. Two algorithms implemented in the Mosel language based on the XPress-suite are presented and compared. The results reveal the relative efficiency of modeling the multi-objective optimization problem using a single-step algorithm, but raise trade-off issues that need further investigations in future work. They also reveal the relevance of using optimal sensor placement in smart parking compared to random topology generation. With the emergence of hybrid networks using unmanned aerial vehicles (UAVs) as communication backbones or communication relays for ground-based sensor networks, a 3D version of our model can be used as the first step towards modeling coverage in hybrid UAV/sensor networks. It can be used, for example, in disaster relief by having emergency spots with priority environmental data to be preferentially relayed by the UAVs or for relieving congested spots or zones of interference in ground-based sensor networks by relaying these spots' data preferentially. This has been reserved for future work. The study of the scalability of the optimized solution when placing a higher number of nodes in a bigger-sized area is another avenue for future research work.

## Figures and Tables

**Table 1:** Static solution: single-step algorithm.

***N***	***N****_S_*	***MaxCov***	***CovCells*_1_**	***Dist*_1_**	***OBJ*_1_**	***UB*_1_**	***GAP*_1_**	***TIME*_1_**
100	5	65	63	14.979	48.021	48.501	1.0%	98
100	6	78	74	18.584	55.416	55.595	0.3%	310
100	7	91	81	21.821	59.179	59.771	1.0%	1297
100	8	104	86	23.821	62.179	66.024	6.2%	3600
100	9	117	90	23.708	66.292	66.954	1.0%	3595
100	10	130	93	24.964	68.036	70.211	3.2%	3600
100	11	143	96	27.579	68.421	69.431	1.5%	3600
100	12	156	98	28.889	69.111	71.643	3.7%	3600
100	13	169	98	30.436	67.564	70.464	4.3%	3600
100	14	182	98	32.537	65.463	70.113	7.1%	3600
100	15	195	99	35.481	63.519	67.356	6.0%	3600
100	16	208	99	39.378	59.622	67.977	14.0%	3600
100	17	221	98	41.477	56.523	65.778	16.4%	3600
100	18	234	99	45.663	53.337	64.624	21.2%	3600
100	19	247	95	48.144	46.856	63.737	36.0%	3600

**Table 2:** Static solution: two-step algorithm.

***N***	***N****_S_*	***MaxCov***	***CovCells*_2_**	***Dist*_2_**	***OBJ*_2_**	***UB*_2_**	***GAP*_2_**	***TIME*_2_**
100	5	65	64	16.215	47.785	47.785	0	3
100	6	78	74	18.584	55.416	55.416	0	10
100	7	91	82	30.190	51.810	51.810	0	21
100	8	104	89	36.673	52.327	52.327	0	11
100	9	117	94	47.169	46.831	46.831	0	14
100	10	130	98	42.664	55.336	55.336	0	22
100	11	143	100	52.470	47.530	47.530	0	13
100	12	156	100	35.894	64.106	64.433	1%	80
100	13	169	100	33.806	66.194	66.523	1%	143
100	14	182	100	34.595	65.405	65.751	1%	633
100	15	195	100	35.643	64.357	64.710	1%	252
100	16	208	100	38.057	61.943	62.322	1%	345
100	17	221	100	43.899	56.101	56.540	1%	1224
100	18	234	100	48.921	51.079	54.322	7%	3600
100	19	247	100	57.094	42.906	51.985	19%	3600

**Table 3:** Comparing static algorithms.

***N****_S_*	**DiffSol (in %)**	**DiffTime (in %)**
5	0.5%	2844%
6	0.0%	2856%
7	14.2%	6140%
8	18.8%	32,328%
9	41.6%	25,181%
10	23.0%	15,612%
11	44.0%	28,370%
12	7.8%	4375%
13	2.1%	2424%
14	0.1%	469%
15	–1.3%	1326%
16	–3.7%	943%
17	0.8%	194%
18	4.4%	0%
19	9.2%	0%

**Table 4:** Comparing adaptive algorithms.

**Parameters**	**Single-Step**	**Two-Steps**
*N_S_*	12	13
*CovCells*	98	100
*Dist*	28.889	33.806
*OBJ*	69.111	66.194
*Upper Bound*	70.988	66.532
*Gap*	2.7%	1%
*Time*	3600s	1635 s
